# Immune responses associated with homologous protection conferred by commercial vaccines for control of avian pathogenic *Escherichia coli* in turkeys

**DOI:** 10.1186/s13567-014-0132-5

**Published:** 2015-01-23

**Authors:** Jean-Rémy Sadeyen, Zhiguang Wu, Holly Davies, Pauline M van Diemen, Anita Milicic, Roberto M La Ragione, Pete Kaiser, Mark P Stevens, Francis Dziva

**Affiliations:** Avian Infectious Diseases Programme, The Pirbright Institute, Compton, Berkshire RG20 7NN UK; The Roslin Institute and Royal (Dick) School of Veterinary Studies, University of Edinburgh, Easter Bush, Midlothian, EH25 9RG UK; The Jenner Institute, University of Oxford, Oxford, OX3 7DQ UK; School of Veterinary Medicine, University of Surrey, Guildford, ᅟ, GU2 7TE UK; Department of Bacteriology, AHVLA, Weybridge, Surrey ᅟ, KT15 3NB UK; Present address: School of Veterinary Medicine, The University of the West Indies, St Augustine, Trinidad and Tobago

## Abstract

Avian pathogenic *Escherichia coli* (APEC) infections are a serious impediment to sustainable poultry production worldwide. Licensed vaccines are available, but the immunological basis of protection is ill-defined and a need exists to extend cross-serotype efficacy. Here, we analysed innate and adaptive responses induced by commercial vaccines in turkeys. Both a live-attenuated APEC O78 Δ*aroA* vaccine (Poulvac® *E. coli*) and a formalin-inactivated APEC O78 bacterin conferred significant protection against homologous intra-airsac challenge in a model of acute colibacillosis. Analysis of expression levels of signature cytokine mRNAs indicated that both vaccines induced a predominantly Th2 response in the spleen. Both vaccines resulted in increased levels of serum O78-specific IgY detected by ELISA and significant splenocyte recall responses to soluble APEC antigens at post-vaccination and post-challenge periods. Supplementing a non-adjuvanted inactivated vaccine with Th2-biasing (Titermax® Gold or aluminium hydroxide) or Th1-biasing (CASAC or CpG motifs) adjuvants, suggested that Th2-biasing adjuvants may give more protection. However, all adjuvants tested augmented humoral responses and protection relative to controls. Our data highlight the importance of both cell-mediated and antibody responses in APEC vaccine-mediated protection toward the control of a key avian endemic disease.

## Introduction

Avian pathogenic *Escherichia coli* (APEC) cause colibacillosis, a complex of respiratory and systemic diseases that exert substantial welfare and economic costs on poultry producers worldwide. Losses are incurred through premature deaths, condemnation of carcasses at slaughter, reduced productivity and recurring costs associated with antibiotic prophylaxis and therapy. A recent longitudinal survey of broiler flocks in the United Kingdom found evidence of colibacillosis in 39% of dead birds [1], and the same authors implicated colibacillosis in 70% of deaths of broiler chicks 2–3 days after placement [2]. A number of risk factors are known, including prior or concurrent infection with respiratory viruses or *Mycoplasma*, stress and injury associated with formation of a social hierarchy, onset of sexual maturity and intense laying, and poor biosecurity, hygiene and ventilation. The control of colibacillosis requires the mitigation of such risk factors, but can also be partly achieved through vaccination.

A major impediment to the design of effective vaccines is the remarkable diversity of *E. coli* associated with avian disease. Though serogroups O1, O2 and O78 and sequence types ST23 and ST95 are commonly observed, it is clear that *E. coli* has evolved to cause avian disease from diverse lineages via the acquisition of distinct virulence genes [3,4]. Indeed, we recently reported that a ST23 serogroup O78 strain differed from the prototype ST95 serogroup O1 strain by over 1100 chromosomal genes and marked variation exists in their plasmid repertoire and content [4-6]. Recent analysis of APEC genome sequences indicates that they may also possess zoonotic potential owing to their similarity to *E. coli* that cause human extra-intestinal infections, such as ascending urinary tract infections, sepsis and neonatal meningitis [7-10]. Of further concern is the emergence of multi-drug resistant *E. coli* strains in poultry, including those encoding extended spectrum beta-lactamases (ESBLs), cephalosporin-resistance and plasmid-mediated quinolone resistance (PMQR) [11-15]. This is compounded by evidence of direct transmission of poultry *E. coli* strains to humans [16,17]. Taken together with the burden of avian disease, a need exists to improve control of APEC in reservoir hosts.

The control of APEC has been largely reliant upon vaccination with autologous bacterins [18-20], but these confer short-lived serotype-specific protection and their effectiveness is blunted by the diversity of *E. coli* capable of infecting poultry. Live-attenuated vaccines are preferable owing to ease of administration and improved cross-serotype protection and hence are entering the market [21,22]. Numerous attenuated mutants have been described and evaluated as candidate live vaccines in experimental models, though direct comparisons of these are lacking and their mode of action is not understood [21,23-25].

In a subacute model of APEC O78 infection in turkeys, we have shown that clearance of primary infection is associated with the induction of both humoral and cell-mediated responses [26]. It is unclear whether such events are also induced by existing commercial vaccines. Passively administered antibody either acquired vertically via egg-yolk [27-29] or administered intravenously [30-32] can be protective against APEC infection. Vaccination of turkeys with a low dose of a live virulent strain appeared to give better protection compared to a heat- or formalin-inactivated non-adjuvanted vaccine [30]. Whilst such protection was associated with humoral responses, innate responses leading to adaptive immunity were not analysed, and the contribution of cell-mediated immunity in protection was not measured. Moreover, the use of a virulent strain or inactivated vaccine without adjuvant does not reflect commercial practice in poultry production. We therefore sought to define the innate and adaptive responses associated with protection conferred by licensed inactivated and live-attenuated vaccines.

## Materials and methods

### Bacterial strains, growth media and preparation of vaccines

*E. coli* serogroup O78:K80 strain EC34195 was isolated from a chicken that died of colibacillosis and has been extensively studied [33]. Deletion of 100 bp of the *aroA* gene of this strain gave rise to the live-attenuated vaccine Poulvac® *E. coli* presently commercialized by Zoetis for the control of avian colibacillosis [22]. The lyophilized Poulvac® *E. coli* vaccine strain was re-constituted in sterile water to ca. 10^9^ colony-forming units (CFU) per mL prior to use. A whole cell formalin-inactivated vaccine based on strain EC34195 was prepared without adjuvant (hereafter designated bacterin), or with a licensed aluminium hydroxide adjuvant, by a supplier of emergency poultry vaccines according to standard procedures (Ridgeway Biologicals Ltd., Compton, UK). A spontaneous nalidixic acid resistant derivative of EC34195 was produced by plating approximately 10 log_10_ colony-forming units (CFU) of EC34195 on MacConkey agar (Oxoid, Basingstoke, UK) supplemented with 25 μg/mL nalidixic acid (Mac + Nal). The subsequent derivative (EC34195nal^R^) was passaged in pure culture and confirmed to possess an identical growth rate, phenotypic characteristics and panel of virulence-associated genes (*cvi/cva*C *iss*, *iuc*D and *tsh*) to the parent strain. Strain EC34195nal^R^ was stored at −70 °C in 15% (v/v) glycerol in Luria Bertani (LB) broth until required. The inoculum of this strain was prepared as described [26].

### Experimental animals

Animal experiments were conducted according to the requirements of the Animal (Scientific Procedures) Act 1986 (licence no. 30/2463) with the approval of the Local Ethical Review Committee. Male Big5FLX turkeys were obtained from Aviagen Ltd. (Tattenhall, Cheshire, UK) as day-old poults, housed and reared as previously described [26].

### Analysis of protection conferred by licensed live-attenuated and whole cell formalin-inactivated APEC O78 vaccines in turkeys

Thirty 1-day old turkey poults were randomly housed in groups of 10 birds per room on floor pens to simulate commercial practice. For the delivery of a coarse aerosol spray of live vaccine, birds were restrained in one corner of the room at a density of 10 birds per 0.25 m^2^, and the vaccine was administered with a syringe fitted with a device that creates a coarse aerosol directed at the faces of individual birds. Group A (control) received 10 mL sterile saline as coarse spray (per group, not individually) while group B received 10 mL of saline containing 10^7^ CFU per mL of the Poulvac® *E. coli* live-attenuated vaccine. Each bird in group C received 0.2 mL (containing the equivalent of approximately 2 × 10^9^ CFU) of the EC34195 inactivated vaccine via the subcutaneous route as per commercial practice and recommended by the manufacturer. Birds were monitored for adverse reactions every 3 h for the first 24 h and thereafter twice daily until the booster vaccinations. Birds in group B were given a booster of ca. 10^7^ CFU per mL in drinking water at 7 days of age. Excess vaccine-treated water was discarded after 18 h. Birds in group C were given a booster (0.4 mL) of the same inactivated vaccine at 14 days of age via the subcutaneous route in the neck region. The control group was given sterile saline in drinking water mixed at the same proportions as for the live vaccine. Birds were monitored twice daily for the remainder of the experiment. On day 41 post-primary vaccination, all the birds were inoculated into the left caudal thoracic airsac with 10^7^ CFU of the EC34195nal^R^ strain, on which both vaccines are based, in 100 μL of saline and monitored overnight for clinical signs. After 24 h, all the birds were killed humanely and subjected to *post-mortem* examination essentially as described [26]. Part of the spleen was collected for T cell re-stimulation assays. Lung, liver, kidney and the remaining spleen tissue were sampled to enumerate tissue-associated bacteria whilst blood and bile were collected for serology.

### Analysis of innate and adaptive responses upon vaccination with licensed live-attenuated and inactivated APEC O78 vaccines in turkeys

Having established vaccine efficacy in pilot studies (above), one hundred and thirty-five 1-day old turkey poults were randomly divided into 3 groups of 45 birds and housed separately. Birds were vaccinated as described above with sterile saline (control group), Poulvac® *E. coli* or inactivated vaccine with aluminium hydroxide adjuvant. Boosters were given at the respective time-points as above. On days 1, 3, 5, 7, 14, 21 and 28 post-primary vaccination, five birds were randomly removed from each group, killed humanely and subjected to *post-mortem* examination. From each bird, sections of liver, lung and spleen were collected in RNAlater (Ambion, Warrington, UK) for cytokine analysis. In the live vaccine group, the remainder of the lung was used to enumerate the vaccine strain as described below. Selected colonies were subjected to PCR analysis spanning the *aroA* gene using the primers (*aroA*-F-TTGAGTTCGAACGTCGTCAC and *aroA*-R-GCAATGTGCCGACGTCTTTG) and agglutination with specific anti-O78 serum (AHVLA, Weybridge, UK) to confirm recovery of the vaccine strain. At day 28 post-primary vaccination, the spleen was collected into ice-cold RPMI1640 medium for use in T cell re-stimulation assays and blood and bile were collected for serology. On day 41 post-primary vaccination, all the remaining birds were challenged, subjected to *post-mortem* examination 24 h later and tissues sampled as described above.

### Total RNA extraction and real-time quantitative RT-PCR for cytokine mRNA

Total RNA extraction and cytokine mRNA analyses were performed essentially as described [26]. Data are expressed in terms of the cycle threshold value (Ct), normalised for each sample using the Ct value of 28S rRNA product for the same sample, as described previously [34,35]. Final results are shown as corrected ΔCt, using the normalised value.

### Enumeration of tissue-associated bacteria

Viable tissue-associated bacteria were enumerated essentially as described [4]. The lower limit of detection by this method was 1.79 log_10_ CFU/g of tissue. Enrichment cultures were also performed in which 1 mL of tissue homogenate was cultured in 9 mL brain heart infusion (BHI) broth for 18 h at 37 °C aerobically, prior to plating on Mac + Nal, giving a theoretical lower limit of detection by enrichment of 1 log_10_ CFU/g of tissue.

### Measurement of antibody and cell-mediated responses

Preparation of APEC soluble antigens for ELISA, quantification of total protein content, storage and subsequent antibody assays were performed essentially as described [26]. Cell-mediated responses were determined by splenocyte proliferation assays again as previously described [26] and data is expressed as mean counts per minute (cpm).

### Analysis of a bacterin mixed with Th1- or Th2-biasing adjuvants

The Th1-stimulating adjuvant CASAC: (**c**ombined **a**djuvant **s**ynergistic **a**ctivation of **c**ellular immunity) was formulated by an adaptation of the method described [36]. Briefly, the first dose comprised 50 μg of monophosphoryl lipid A (MPL; Invivogen, Toulouse, France), 50 μg of Pam3CysSerLys4 (Pam3CSK4; EMC Microcollections, Tuebingen, Germany) and 50 μg of chicken interferon-γ (2B Scientific**,** Upper Heyford, UK) homogenized with 100 μL (same dose as previously used) of formalin-inactivated EC34195 supplied to commercial specification by Ridgeway Biologicals Ltd. but without adjuvant (bacterin). The booster comprised the same ingredients of CASAC mixed with 200 μL of bacterin. Bacterin formulated with a second Th1-biasing adjuvant was separately prepared using synthetic cytosine phosphodiester-guanine (CpG)-containing oligodeoxynucleotides (ODN) as described [37]. The sequence for CpG-ODN, TC**GTCGTT**T**GTCGTT**TT**GTCGTT** (bold motifs enhance CpG-ODN activity), was synthesized with a phosphothionate backbone (Sigma). The first dose was prepared by mixing 20 μg of synthetic CpG-ODN (in sterile pyrogen-free water) with 100 μL of the non-adjuvanted EC34195 bacterin. The booster dose comprised the same amount of CpG-ODN in 200 μL of non-adjuvanted EC34195 bacterin.

For comparison, the formalin-inactivated bacterin without adjuvant was also formulated with Th2-stimulating adjuvants. The first of these comprised a dose of 100 μL of EC34195 bacterin thoroughly mixed with an equal volume (ratio of 1:1) of Titermax® Gold (Sigma) as per manufacturer’s instructions. The booster dose was 200 μL of the homogenous mixture. EC34195 bacterin formulated with aluminium hydroxide adjuvant was also tested as another Th2-stimulating adjuvant; 100 μL for primary vaccination and 200 μL for the booster (same dose as in the pilot studies).

For vaccination, thirty 1-day old turkey poults were randomly divided into 6 groups of 5 birds and housed separately as before. Group 1 received sterile saline (negative control group), group 2 received formalin-inactivated EC34195 bacterin without adjuvant (control group), group 3 received the same bacterin formulated with CpG-ODN, group 4 received the same bacterin with CASAC, group 5 received the same bacterin mixed with aluminium hydroxide, group 6 received the same bacterin mixed with Titermax® gold. All vaccines were administered by the subcutaneous route with booster doses for the respective vaccine formulations given at 14 days of age via the same route. Birds were monitored twice daily for the duration of the experiment. On day 41 post-primary vaccination, all the birds were inoculated with 10^7^ CFU of strain EC34195nal^R^ in 100 μL of saline into the left caudal thoracic airsac as above and monitored overnight for clinical signs. All the birds were killed humanely and subjected to *post-mortem* examination and collection of organs and blood as above.

### Statistical analyses

Cytokine data was analysed for statistical significance using a one-way ANOVA test with an *ad hoc* Tukey’s test. Bacterial counts recovered from tissues, antibody levels and splenocyte recall responses were analysed by Student’s *t*-test. *P* values ≤ 0.05 were considered significant.

## Results

### Licensed live-attenuated and formalin-inactivated vaccines based on APEC O78 are protective against homologous challenge in turkeys

To evaluate the protective efficacy of existing vaccines and establish models in which the basis of protection can be dissected, turkeys were vaccinated using Poulvac® *E. coli* or an inactivated vaccine based on the parent strain of Poulvac® *E. coli* and compared to mock-vaccinated turkeys. In birds given the live vaccine, up to ca. 4 log_10_ CFU per gram lung tissue of the vaccine strain was detected 1, 3, 5 and 7 days post-primary vaccination (data not shown). Recovery of the vaccine strain was confirmed by a specific PCR for the *aroA* gene in *E. coli* isolated from the lung homogenates at these times (data not shown). The live-attenuated vaccine strain was not detectable at any other time-points in any group.

**Figure 1:**
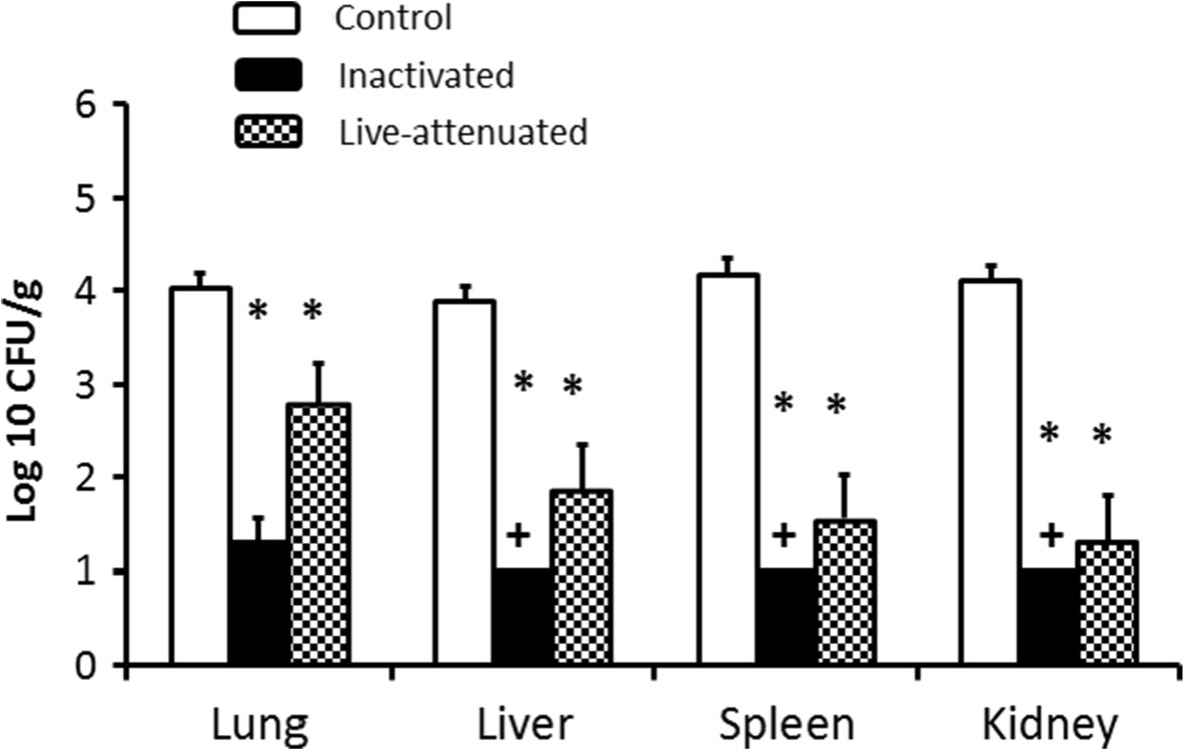
**Recoveries of strain EC34195nal** ^**R**^ **from organs of vaccinated turkeys at 24 h post-challenge.** Turkey poults were given sterile saline (mock) as a coarse spray (open bars), a formalin-inactivated vaccine of the parent strain EC34195 formulated in aluminium hydroxide adjuvant via the subcutaneous route (black bars) or Poulvac® *E. coli* as a coarse spray (checked black and white bars) starting at one day-old as indicated in the Materials and methods. All turkeys were challenged via the intra-airsac route at day 41 post-primary vaccination with EC34195nal^R^ and the indicated tissues recovered 24 h later. The data are expressed as mean log_10_ CFU/g tissue ± standard error of the mean. The limit of detection by direct plating was 1.79 and limit of detection by enrichment was 1 log_10_ CFU/g tissue; + denotes values after enrichment of tissue homogenates. *denotes *P* ≤ 0.05 relative to mock-vaccinated controls.

Upon intra-airsac challenge of vaccinated birds with a nalidixic acid resistant derivative of the strain used to prepare the vaccines, significant protection was observed in the groups that received either Poulvac® *E. coli* or the inactivated vaccine (Figure 1). Approximately 4 log_10_ CFU/g EC34195nal^R^ were recovered from the lung, liver, spleen and kidney of mock-vaccinated turkeys. In contrast, nalidixic acid resistant *E. coli* were only detectable in the spleens, livers and kidneys of turkeys that received the inactivated vaccine by enrichment, where the theoretical lower limit of detection was 1 log_10_ CFU/g (Figure 1; *P* < 0.05). The number of nalidixic acid resistant *E. coli* recovered from the lung, liver, spleen and kidney of turkeys that received the live vaccine was significantly reduced by around 2 log_10_ CFU/g (Figure 1; *P* < 0.05). Consistent with the lower numbers of bacteria recovered from the organs of vaccinated birds after challenge with EC34195nal^R^, none of the vaccinated birds exhibited clinical signs or pathological lesions indicative of colibacillosis as previously defined [26]. By contrast, all mock-vaccinated birds challenged with EC34195nal^R^ exhibited clinical signs typical of colibacillosis (respiratory distress, hunched posture, ruffled feathers, reduced response to stimulus) and gross *post-mortem* lesions including airsacculitis, pericarditis, perihepatitis and fibrin deposits on serosal surfaces at 24 h post-challenge. It can therefore be inferred that the strain is virulent and that live-attenuated or inactivated vaccines derived from it confer protection against homologous re-challenge.

### Quantification of cytokine transcripts indicates that licensed live-attenuated and inactivated APEC O78 vaccines predominantly induce a Th2 response in the spleen of turkeys

To assess cytokine responses as signatures of innate and adaptive immune responses induced by the live-attenuated and inactivated vaccines, turkey poults were vaccinated as above and killed at intervals. We analysed the levels of transcripts encoding a pro-inflammatory cytokine (IL-1β), a pro-inflammatory chemokine (CXCLi2), a signature Th1 cytokine (IFN-γ), a signature Th2 cytokine (IL-13) and two signature T regulatory cytokines (IL-10 and TGF-β4), at intervals post-primary and -secondary vaccination.

**Figure 2:**
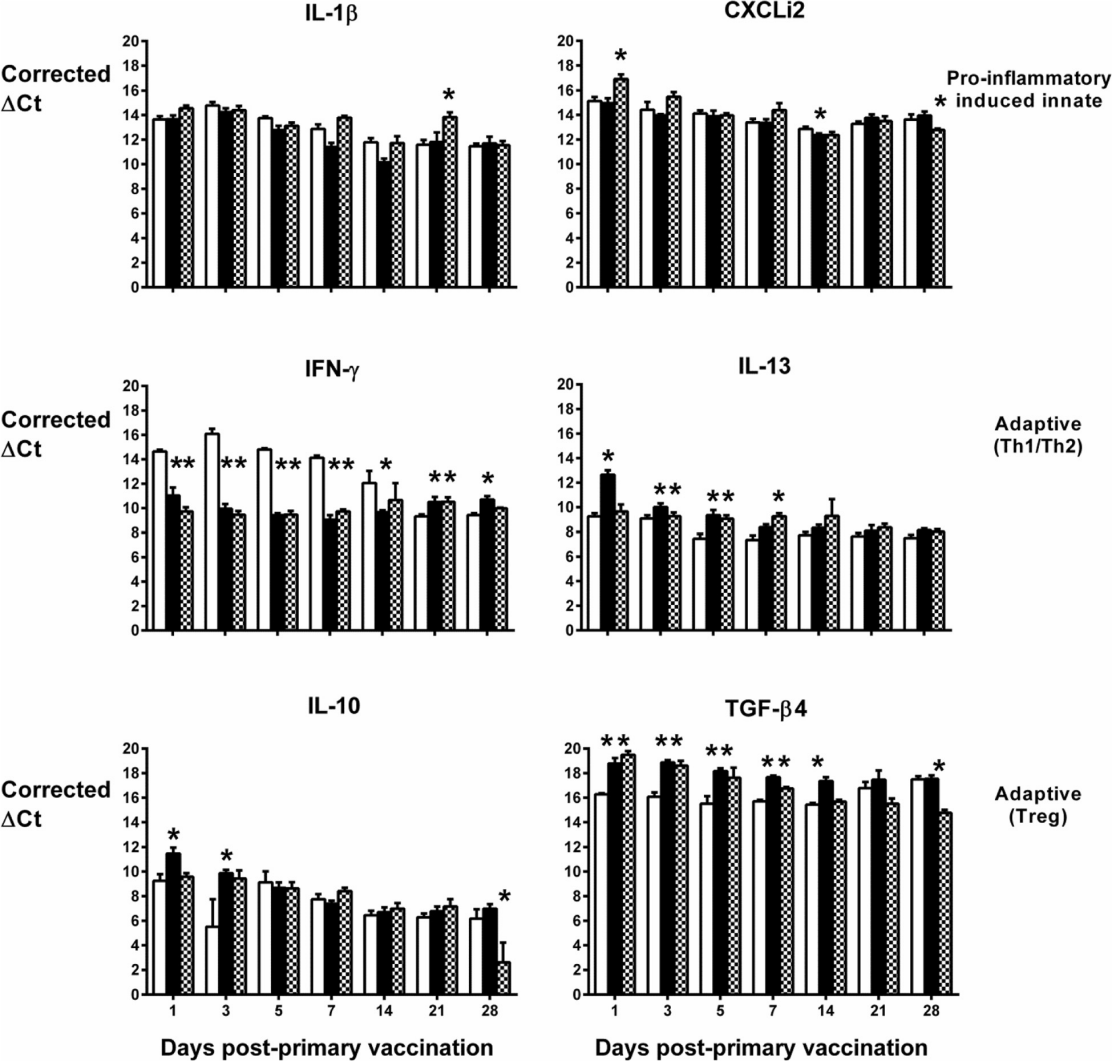
**Levels of cytokine transcripts in the spleens of vaccinated turkeys as measured by real-time qRT-PCR.** Results are expressed as corrected ΔCt values ± standard error of the mean after normalization with the Ct value of 28S rRNA product of each sample. Turkey poults were given sterile saline (mock) as a coarse spray (open bars), a formalin-inactivated vaccine based on the parent strain EC34195 formulated in aluminium hydroxide adjuvant via the subcutaneous route (black bars) or Poulvac® *E. coli* as a coarse spray (checked black and white bars) starting at one day-old. Five birds from each group were sampled per time-point. *denotes *P* ≤ 0.05 relative to mock-vaccinated controls.

**Figure 3:**
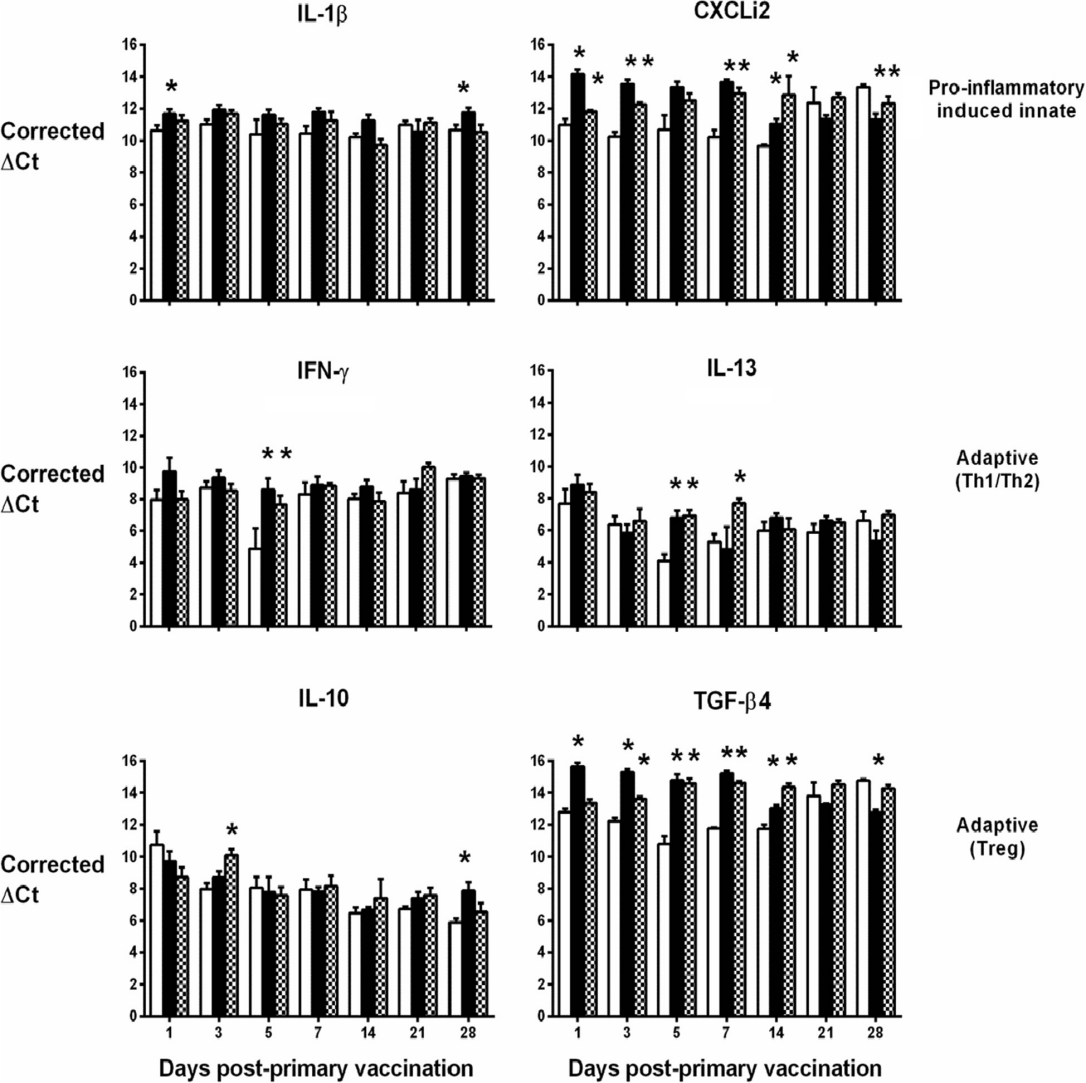
**Levels of cytokine transcripts in the livers of vaccinated turkeys as measured by real-time qRT-PCR.** Details are as in the legend to Figure 2.

**Figure 4:**
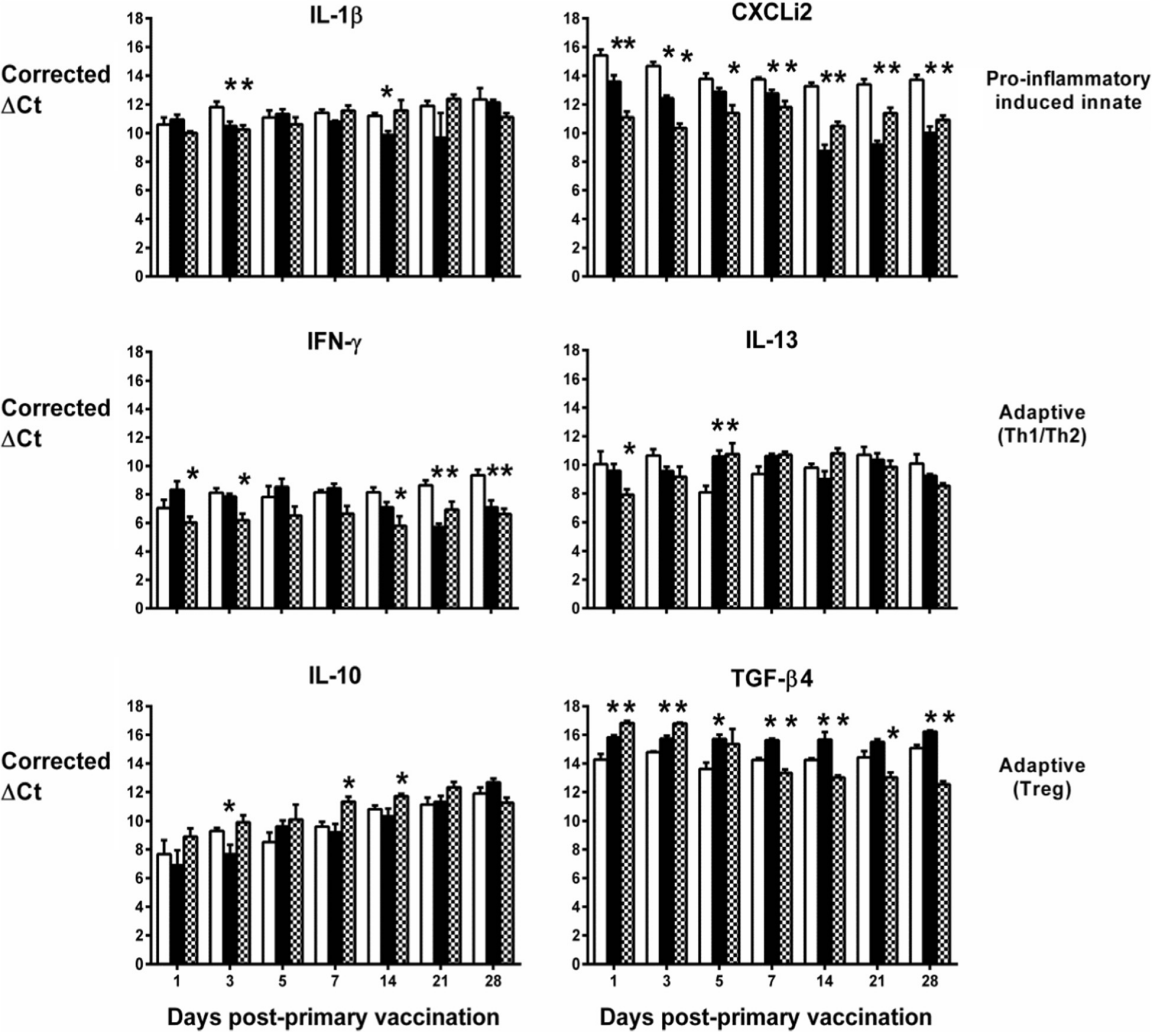
**Levels of cytokine transcripts in the lungs of vaccinated turkeys as measured by real-time qRT-PCR.** Details are as in the legend to Figure 2.

The normalised levels of transcripts encoding these cytokines are shown over time in the spleen (Figure 2), liver (Figure 3) and lung (Figure 4). In the spleen, the live-attenuated and inactivated vaccines significantly induced transcription of IL-13 in the first week post-primary vaccination (Figure 2; *P* < 0.05). In the same period, transcription of the signature Th1 cytokine IFN-γ was significantly repressed following vaccination relative to levels in the mock-vaccinated controls (Figure 2; *P* < 0.05), and transcription levels of the anti-inflammatory cytokine TGF-β4 (the chicken equivalent of mammalian TGF-β1) were significantly induced following vaccination (Figure 2; *p* < 0.05). Together, these data indicate a bias towards a Th2 response at a key site of immune priming during the typical period of induction of an adaptive immune response to primary vaccination.

In the other two organs, the pattern is less clear. Elevated IL-13 mRNA expression levels were also detected in the liver (Figure 3) and lung (Figure 4), but only at a couple of time-points and with no discernible pattern in the first week post-primary vaccination with live-attenuated or inactivated vaccines. In the lung, IFN-γ transcription was generally lower in turkeys given the live-attenuated vaccine compared to mock-vaccinated birds, significantly so at multiple time-points (Figure 4). However, the inactivated vaccine only significantly repressed IFN-γ transcription in the lung at 21 and 28 days post-primary vaccination, time-points which would represent the resolution of a normal adaptive immune response to primary vaccination (Figure 4). No repression of IFN-γ transcription was detected in the liver at any time-point (Figure 3). In both organs, we detected significant induction of TGF-β4 mRNA expression in turkeys vaccinated with the live-attenuated or inactivated vaccines, particularly in the first two weeks post-primary vaccination (Figures 3 and 4; *P* < 0.05).

Differences between groups in transcription of the other cytokines analysed did not show consistent trends across organs or time in vaccinated turkeys relative to controls, with the exception of CXCLi2 expression, which was unaltered following vaccination in the spleen (Figure 2), consistently down-regulated in the lung (Figure 4; *P* < 0.05) and generally up-regulated in the liver (Figure 3; *P* < 0.05). CXCLi2 mainly chemo-attracts monocytes [35], and this differential expression may reflect traffic of these cells in response to vaccination.

### Vaccine-induced protection against homologous challenge is associated with elevated adaptive responses

**Figure 5:**
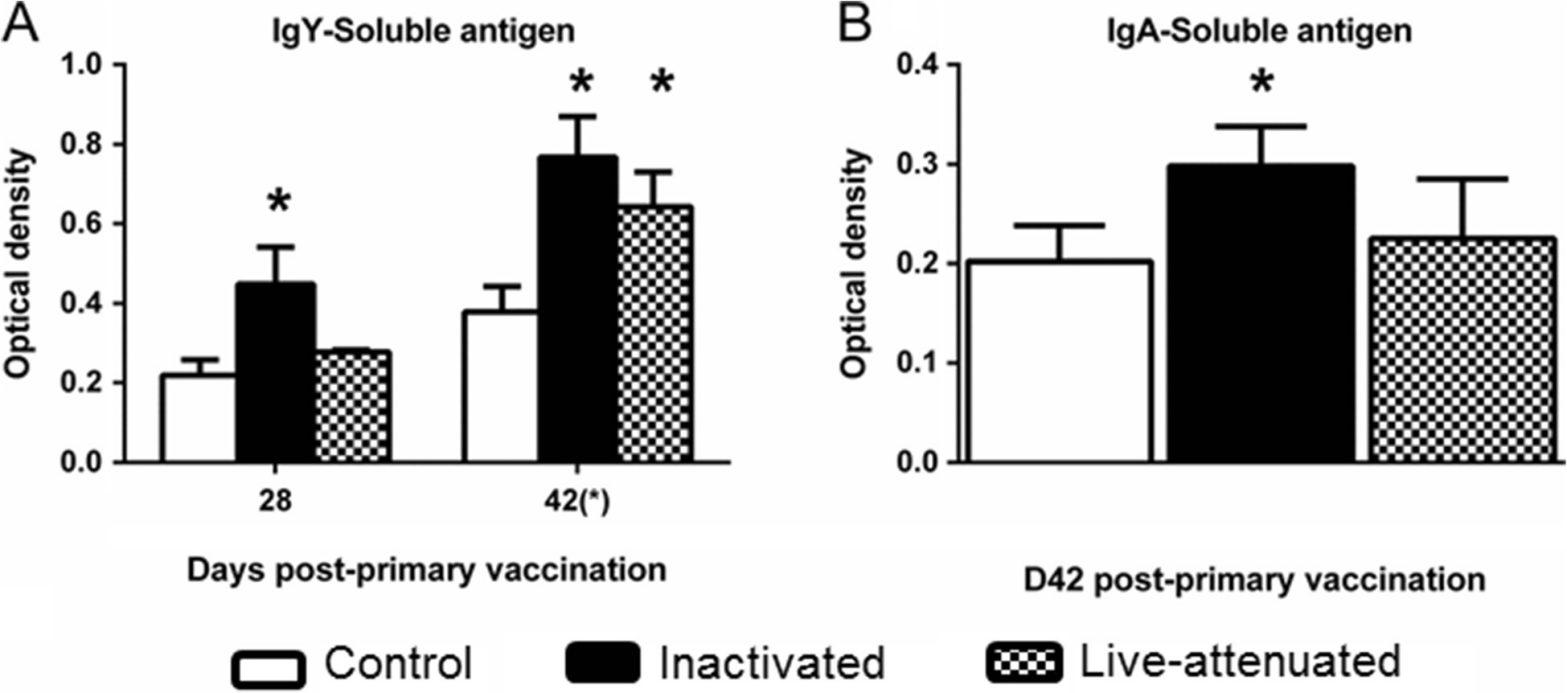
**APEC-specific antibody responses in vaccinated turkeys before and after challenge.** Turkey poults were given sterile saline (mock) as a coarse spray (open bars), a formalin-inactivated vaccine based on the parent strain EC34195 formulated in aluminium hydroxide adjuvant via the subcutaneous route (black bars) or Poulvac® *E. coli* as a coarse spray (checked black and white bars) starting at one day-old. Five birds from each group were sampled at day 28 post-primary vaccination and 10 birds per group at day 42 post-primary vaccination (24 h after intra-airsac challenge with EC34195nal^R^) and serum IgY **(A)** and bile IgA **(B)** levels determined by an APEC-specific ELISA. *denotes *P* ≤ 0.05 relative to mock-vaccinated controls.

To identify adaptive immune responses associated with protection against homologous challenge induced by each vaccine, we measured humoral and cellular responses at intervals after primary and secondary vaccination of turkeys using samples from the same birds as used for cytokine analysis. Immunoglobin Y (IgY) levels and splenocyte proliferation responses were measured in birds killed at day 28 and after challenge (day 42) whereas IgA in bile was measured 24 h after challenge. Both vaccines induced elevated IgY levels post-primary vaccination relative to the mock-vaccinated group, for the inactivated vaccine at both intervals and for the live-attenuated vaccine at day 42 (Figure 5A; *P* ≤ 0.05). APEC-specific IgA was elevated in bile from poults given the inactivated vaccine relative to the mock-vaccinated group (Figure 5B; *P* ≤ 0.05).

**Figure 6:**
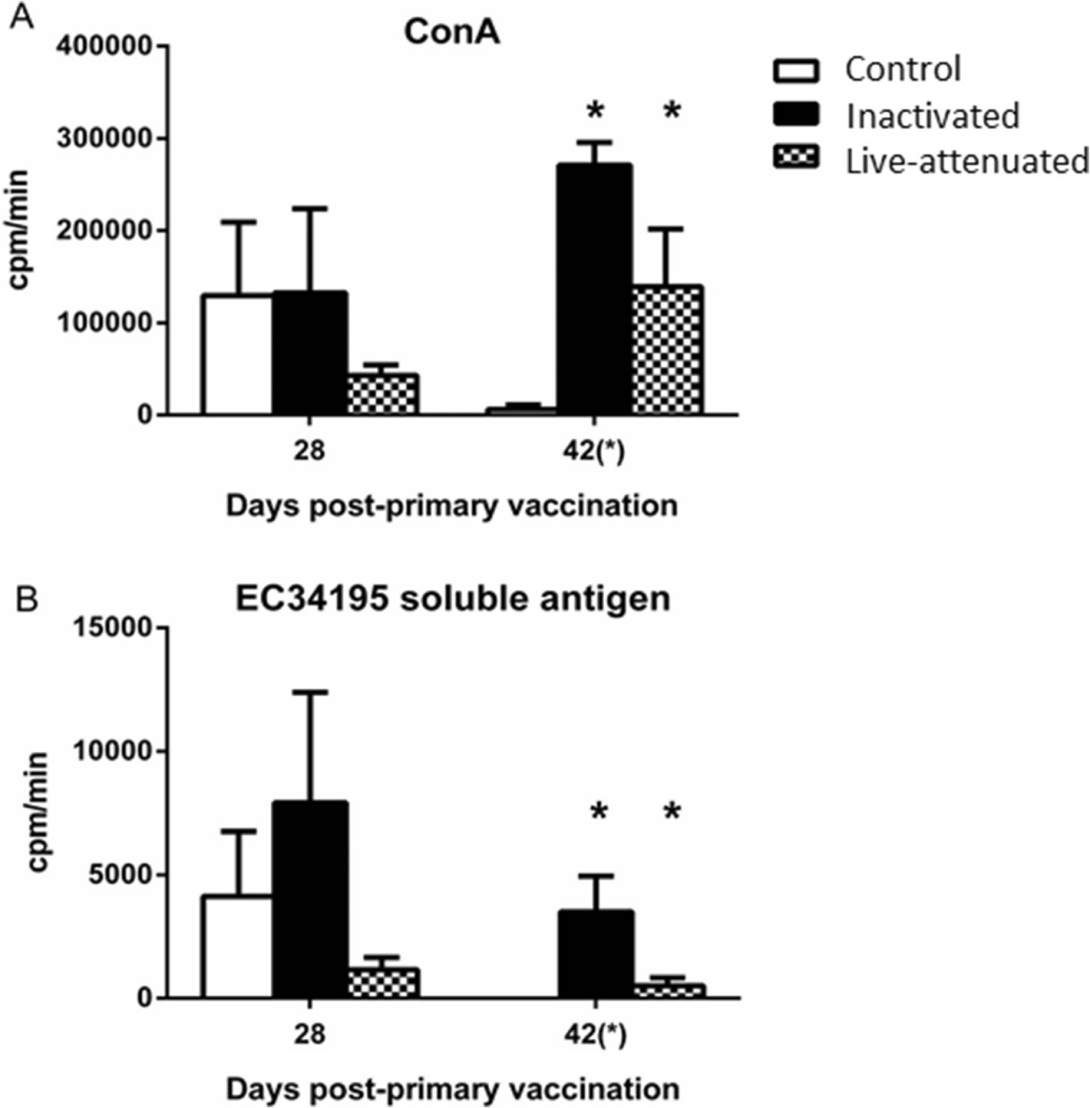
**Turkey splenocyte proliferation responses to Concanavalin A and EC34195 soluble lysate in vaccinated turkeys before and after challenge.** Turkey poults were given sterile saline (mock) as a coarse spray (open bars), a formalin-inactivated vaccine based on the parent strain EC34195 formulated in aluminium hydroxide adjuvant via the subcutaneous route (black bars) or Poulvac® *E. coli* as a coarse spray (checked black and white bars) starting at one day-old. Five birds were sampled from each group at each time and splenocytes re-stimulated with ConA **(A)** or EC43195 soluble lysate **(B)**. The data are presented as mean counts per minute ± standard error of the mean. *denotes *P* ≤ 0.05 relative to mock-vaccinated controls.

The ability of splenocytes recovered from each group to proliferate in response to mitogen or soluble EC34195 antigen was also evaluated at day 28 post-primary vaccination and at *post-mortem* examination on day 42 (24 h after challenge). The response to ConA was comparable between the birds given mock- or inactivated vaccine at day 28, and was not significantly different from control birds in the group given Poulvac® *E. coli* (Figure 6A). After challenge, almost no proliferation of splenocytes from the control group was seen in response to ConA (Figure 6A), consistent with a suppressive effect of APEC O78 infection detected following primary and secondary infection [26]. Splenocytes recovered from birds given live-attenuated or inactivated vaccines at day 42 proliferated in response to ConA (Figure 6A).

Splenocyte proliferation in response to APEC soluble antigen was significantly elevated both vaccinated groups at day 42 relative to levels in the mock-vaccinated group (Figure 6B, *P* < 0.05). Responses were lower in the group given the live-attenuated vaccine relative to the control group at day 28 and relative to the inactivated vaccine group at both time-points. At day 42, no response to EC34195 soluble antigen was seen in the control group.

### Impact of Th1- and Th2-stimulating adjuvants on protection conferred by the APEC O78 inactivated bacterin

To examine the effects of Th1- and Th2-stimulating adjuvants, we formulated the EC34195 inactivated bacterin with two compounds with a reported bias toward Th1 responses (CASAC or CpG) and two with a reported bias to Th2 responses (aluminium hydroxide or TiterMax®Gold) [36,38,39]. The vaccines were given to day-old turkey poults and again 14 days later by the subcutaneous route, with intra-airsac challenge with EC34195nal^R^ at day 41, as described in pilot studies where protection conferred by the inactivated vaccine was shown (Figure 1). A bacterin without adjuvant and mock-vaccination with saline were used as controls.

**Figure 7:**
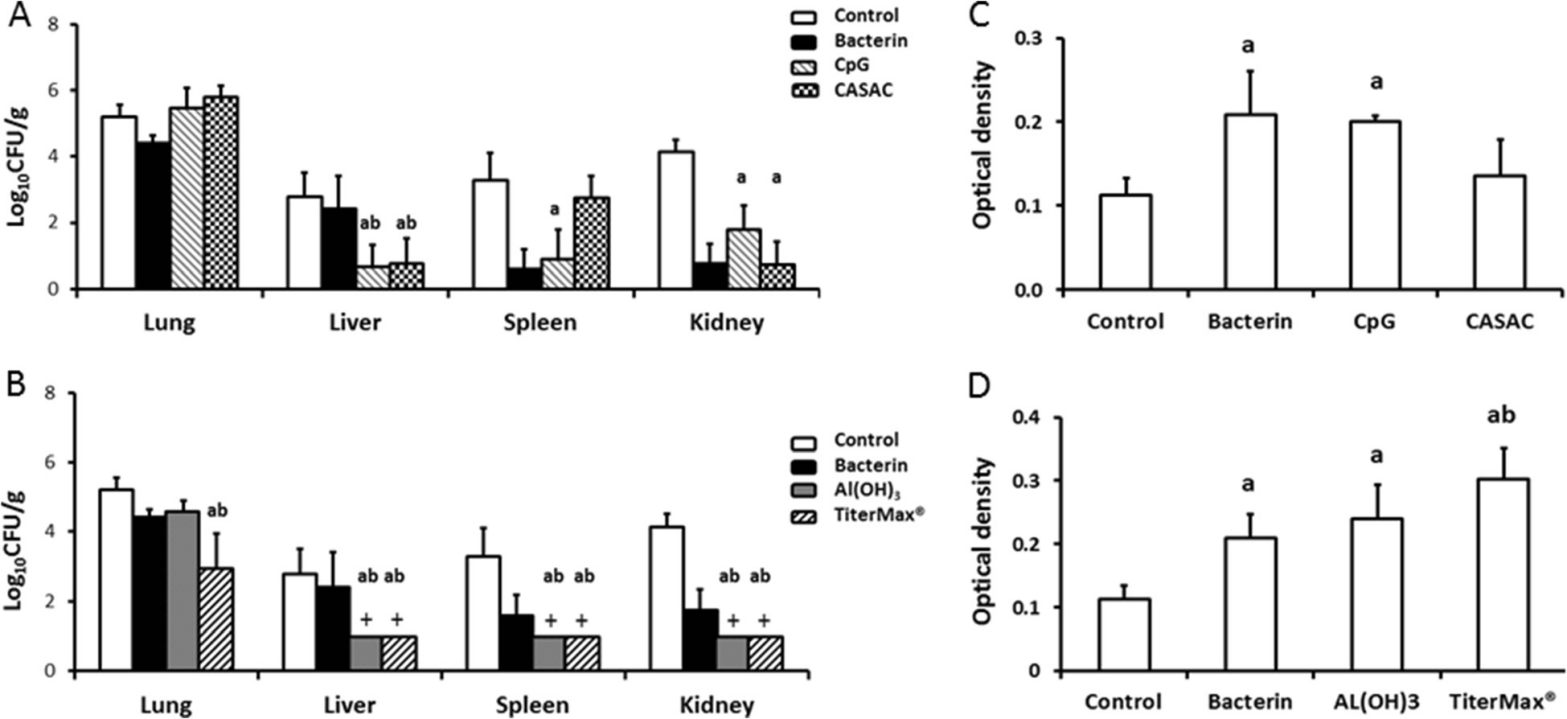
**Recoveries of strain EC34195nal** ^**R**^ **from organs of turkeys vaccinated with a formalin-inactivated bacterin formulated with different adjuvants at 24 h post-challenge.** Turkey poults were given sterile saline (negative control), an EC34915-based inactivated bacterin without adjuvant, or formulated with adjuvants that promote a Th1 response (CASAC or CpG-oligonucleotides **(A)**), or Th2 response (Titermax® Gold or aluminium hydroxide **(B)**). All vaccines were given by the subcutaneous route at 1 day-old and boosted at day 14. All the birds were challenged at day 41 post-primary vaccination via the intra-airsac route with EC34195nal^R^ and sampled after 24 h. The data are presented as mean log_10_ CFU/g tissue ± standard error of the mean. APEC-specific serum IgY levels for corresponding birds at post-mortem examination were measured by ELISA compared to mock- and bacterin-vaccinated groups, Th1- **(C)** and Th2-stimulating adjuvants **(D)**. + denotes value of EC34195nal^R^ after enrichment. “a” denotes *P* ≤ 0.05 relative to turkey poults that received sterile saline (mock-vaccinated group), “b” denotes *P* ≤ 0.05 relative to bacterin alone, and “ab” is significance relative to both mock- and bacterin-vaccinated groups.

At 24 h post-challenge, statistically significant reductions in the numbers of EC34195nal^R^ recovered from the liver and kidney were observed in birds vaccinated with bacterin formulated with either CASAC or CpG oligonucleotides as adjuvants, and in the spleen of birds vaccinated with CpG oligonucleotides, relative to levels in the mock-vaccinated group (Figure 7A; *P* < 0.05). In the groups that received the bacterin formulated with CASAC or CpG, there was also a significant reduction in bacterial numbers in the liver relative to the group that received the bacterin alone, but this difference was not seen in the spleen, kidney or lung (Figure 7A).

In the turkeys vaccinated with the bacterin formulated with the Th2-stimulating adjuvants aluminium hydroxide or TiterMax®, no nalidixic acid resistant *E. coli* were isolated from the liver, kidney and spleen on direct plating (Figure 7B), but they were detected by enrichment of homogenates of these organs (a theoretical limit of detection of 1 log_10_ CFU/g). Differences in bacterial numbers were statistically significant in all organs from the group that received bacterin formulated with TiterMax® relative to either the bacterin alone or mock-vaccinated control groups (*P* < 0.05). Significant reductions in bacterial numbers were also seen in the liver, spleen and kidney of birds that received the bacterin with aluminium hydroxide relative to the groups that received mock, bacterin alone, or bacterin formulated with CASAC or CpG (Figure 7A and B).

On analysis of APEC-specific IgY at 42 days post-primary vaccination, the bacterin alone induced significantly elevated APEC-specific IgY levels relative to mock vaccinated birds (Figure 7C; *P* < 0.05). The magnitude of the APEC-specific IgY response was no greater when the bacterin was formulated with CpG and marginally lower when formulated with CASAC relative to bacterin alone (Figure 7C). Levels of APEC-specific IgY were no greater in the groups that received the bacterin formulated with aluminium hydroxide and marginally greater when formulated with TiterMax® compared to the bacterin alone (Figure 7D). There is therefore a suggestion that these Th2-biasing adjuvants might generate greater levels of APEC-specific IgY response than Th1-biasing adjuvants.

## Discussion

We recently analysed innate and adaptive responses induced by primary APEC O78 infection and demonstrated an association of these with protection against homologous re-challenge [26]. Here, we extended our studies to dissect the immune responses induced by licensed APEC O78-based live-attenuated and inactivated vaccines and their association with protection. We verified that the two vaccines (Poulvac® *E. coli* and a formalin-inactivated vaccine based on its parent strain prepared to commercial specifications) were protective against homologous intra-airsac challenge with EC34195nal^R^ of turkeys (Figure 1) before examining vaccine-induced innate and adaptive responses. Protection was observed both at the level of clinical signs and the appearance of gross *post-mortem* lesions typical of colibacillosis, as well as the burden of the challenge strain in the lungs and visceral organs. Whilst we were reliably able to detect vaccine-mediated protection against systemic disease and lung colonisation in the turkey model, we acknowledge that it relies on homologous challenge with high doses instilled directly into the left caudal airsac. The basis of protection may therefore differ when turkeys are challenged by heterologous APEC via other routes or natural exposure, and indeed may differ in other avian species. We also acknowledge that the protection observed is largely against bacterial replication in internal organs after systemic translocation of APEC rather than at mucosal surfaces, as the model mimics the often fatal form of avian colibacillosis but not superficial or subacute infections where limited invasion occurs.

Variance between the birds receiving the live-attenuated vaccine by coarse spray and in the drinking water was limited, implying that reasonably uniform doses were given despite the inherent limitations of the mode of delivery. We recovered consistent numbers of the Poulvac® *E. coli* strain from the lung and internal organs of vaccinated birds, and confirmed isolates had the expected genotype. The protection observed is consistent with a recent study by the producers of the vaccine strain [22]. Although vaccination of birds less than 2 weeks of age against *E. coli* can lead to poor protection, which is thought to be due to the relative immaturity of their immune system at that point [27], our protocol adhered to the recommended practice described for Poulvac® *E. coli* [22], and we believe that the protective responses observed in our models are such that an evaluation of the role of innate and adaptive responses in the protection observed is justified.

Inactivated vaccines based on formalin- or heat-inactivated *E. coli* are generally believed to confer protection against avian colibacillosis in an antibody-dependent manner [30,40]. This has been partly established through passive transfer of hyper-immune serum [29-32] or egg-yolk antibody [27,28,41]. However, cytokine responses to APEC vaccination have not previously been analysed. Similarly, whilst live-attenuated mutants of various kinds can confer resistance against APEC in target host species [21,22,30,42-44], the innate and cell-mediated responses they elicit are ill-defined. Studies by Fernandez Filho et al. [43] with Poulvac® *E. coli* only examined peripheral cellular responses to vaccination, with a fine spray, of day-old chicks. No booster was given to those birds at 7 days of age via drinking water as is recommended [22]. A recent field trial of Poulvac® *E. coli* [42] also supports that giving a booster in drinking water is not necessary. Interestingly, a higher level of peripheral B lymphocytes was detected in the control than the vaccinated group and the authors speculated that Poulvac® *E. coli* may suppress humoral responses and that they may therefore be dispensable in protection [43]. In support of this, Salehi et al. [44] also reported a lack of significant induction of antibody response in chickens after aerosol administration of an independent *aroA* mutant of *E. coli*. In contrast, we observed that Poulvac® *E. coli* elicited significantly elevated APEC-specific serum IgY in turkeys relative to mock-vaccinated poults. Differences between this study and earlier reports could of course be due to variations in the vaccination protocols, in particular the administration of single dose of the vaccine when birds are still immunologically immature.

The induction of APEC-specific humoral responses by both vaccines in this study is supported by analysis of transcription of cytokines and chemokines at a key site of immune priming, the spleen. In the spleen, during the first week post-vaccination, transcription of the signature Th1 cytokine IFN-γ was repressed, whereas that of the signature Th2 cytokine IL-13 was significantly induced by both vaccines. Reduced transcription of IFN-γ was also seen in the lung at multiple time-points after vaccination with the live-attenuated vaccine but was not evident in the liver. IL-13 mRNA expression levels in the lung and liver in the first week post-primary vaccination showed no discernible pattern. The expression of TGF-β4 in all organs could be suggestive of a proliferative cellular response that requires some kind of regulation. Transient expression of IL-6 was observed in the first 5 days in the spleen, but not liver or lung (data not shown). Since the nature of such proliferative cellular responses could not be discerned, we reasoned that TGF-β4 acted primarily as an anti-inflammatory suppressing the expression of IL-6 in all organs. Overall, the data are indicative of a Th2-biased response to vaccination in the spleen, albeit this is less evident in the liver and lung. The magnitude of cytokine responses was not markedly different between the live-attenuated and inactivated vaccine groups and therefore cannot readily be associated with the magnitude of the adaptive responses measured.

After challenge, splenocyte recall responses to ConA or APEC-specific antigens in the control group (with a high bacterial recovery in the spleen) were poor and absent respectively. These data are consistent with the suppressive effect of APEC O78 on proliferative responses detected in our earlier studies [26], and may imply that the live-attenuated vaccine also has a negative effect on the capacity of splenocytes to proliferate in response to mitogen. As the live-attenuated vaccine could not be recovered from vaccinated turkey poults at day 28, it is difficult to separate the extent to which proliferative responses are an accurate reflection of recall responses, or were compromised owing to presence of low numbers of bacteria or their products. The possibility of suppressive effects makes it difficult to interpret the response in the group given the inactivated vaccine at day 42, as the spleen was recovered after challenge of such birds. Earlier evidence suggests that inactivated vaccines are poor inducers of T cell-mediated immunity [45]. However, we detected significantly elevated splenocyte proliferative responses to APEC-specific antigens induced by the inactivated vaccine after challenge. Studies with a killed *Salmonella* vaccine have also shown a significant induction of lympho-proliferative responses, though these were age- and antigen-dependent [46]. APEC membrane vesicles can evoke both antibody production and T cell proliferation and specifically stimulate T cytotoxic cells to confer resistance against APEC [47], further indicating that inert APEC vaccines can stimulate both adaptive pathways.

Given the potential importance of the Th2 pathway in protection conferred by the vaccines tested, we evaluated the effects of Th1- and Th2-stimulating adjuvants using an EC34195-based bacterin. This enabled us to standardize the dose and timing and compare protection observed without adjuvant with that given by bacterin formulated with aluminium hydroxide that had already proven to be protective throughout. Although the use of Th1 adjuvants resulted in significant reduction in bacterial numbers recovered from internal organs relative to the control (mock-vaccinated) group, the Th2 adjuvants elicited better protective responses in the lungs (especially TiterMax®) and internal organs. CpG-ODN induce predominantly a Th1 response [38,39,48], and administration of these on their own 3 days prior to challenge confers protection against *E. coli* infections in neonatal and adult chickens [49,50]. Furthermore, formulating an inactivated vaccine with these motifs imparted a more potent effect in halting the spread of a lethal APEC strain [51], implying the importance of Th1 responses in resistance against APEC. In this study CpG-ODN did not significantly augment humoral responses or protection relative to the bacterin alone, except in the liver. CASAC is a recently described potent Th1-stimulating adjuvant consisting of three components [36], including interferon-gamma (IFN-γ). We used chicken IFN-γ owing to its commercial availability, and is biologically cross-reactive in the turkey [52]. As with CpG, CASAC did not potentiate humoral responses or protection relative to those seen with the bacterin alone, except in the liver. One of the Th2-biasing adjuvants (TiterMax®) gave significantly improved protection relative to the bacterin alone as well as significantly elevated APEC-specific IgY levels, whereas the other (aluminium hydroxide) was not different to responses to the bacterin alone, in accordance with earlier reports [18,20]. While it is not conclusively clear if Th2 adjuvants stimulate more potent antibody responses and protection than Th1 adjuvants in the context of inactivated APEC vaccines, the data suggest that further research to augment Th2 responses has merit.

In conclusion, we show that live and inactivated APEC vaccines are protective against experimental intra-airsac challenge in a turkey model of acute colibacillosis and that they both induce predominantly a Th2 response in the spleen that correlates with elevated APEC-specific antibody levels. Whilst we have defined the responses associated with vaccine-mediated protection against homologous challenge, the relative contribution of humoral and cell-mediated immunity in protection against heterologous APEC infection requires further study. Demonstration of a key role for antibody will help to prime reverse vaccinology to identify conserved surface-exposed constituents of APEC that can elicit antibody to fix complement and prime phagocytosis. Although our recent sequencing of APEC indicates that *E. coli* evolve to cause avian disease from varied lineages via acquisition of distinct sets of virulence genes, we also revealed a core genome encoding of over 3000 conserved factors [4]. Mining of the core genome of human extraintestinal pathogenic *E. coli* strains has yielded a subset of factors that show promise as cross-protective vaccines for control of sepsis and urinary tract infections [53,54], and a similar approach for avian colibacillosis has merit. The models and data presented here will help to inform and evaluate improved vaccines in target hosts.
